# Alternative Splicing of NURF301 Generates Distinct NURF Chromatin Remodeling Complexes with Altered Modified Histone Binding Specificities

**DOI:** 10.1371/journal.pgen.1000574

**Published:** 2009-07-24

**Authors:** So Yeon Kwon, Hua Xiao, Carl Wu, Paul Badenhorst

**Affiliations:** 1 Institute of Biomedical Research, University of Birmingham, Edgbaston, United Kingdom; 2 Laboratory of Biochemistry and Molecular Biology, National Cancer Institute, National Institutes of Health, Bethesda, Maryland, United States of America; European Molecular Biology Laboratory, Germany

## Abstract

*Drosophila* NURF is an ISWI–containing chromatin remodeling complex that catalyzes ATP–dependent nucleosome sliding. By sliding nucleosomes, NURF can alter chromatin structure and regulate transcription. NURF301/BPTF is the only NURF–specific subunit of NURF and is instrumental in recruiting the complex to target genes. Here we demonstrate that three NURF301 isoforms are expressed and that these encode functionally distinct NURF chromatin remodeling complexes. Full-length NURF301 contains a C-terminal bromodomain and juxtaposed PHD finger that bind histone H3 trimethylated at Lys4 (H3K4me3) and histone H4 acetylated at Lys16 (H4K16Ac) respectively. However, a NURF301 isoform that lacks these C-terminal domains is also detected. This truncated NURF301 isoform assembles a complex containing ISWI, NURF55, and NURF38, indicating that a second class of NURF remodeling complex, deficient in H3K4me3 and H4K16Ac recognition, exists. By comparing microarray expression profiles and phenotypes of null *Nurf301* mutants with mutants that remove the C-terminal PHD fingers and bromodomain, we show that full-length NURF301 is not essential for correct expression of the majority of NURF gene targets in larvae. However, full-length NURF301 is required for spermatogenesis. Mutants that lack full-length NURF exhibit a spermatocyte arrest phenotype and fail to express a subset of spermatid differentiation genes. Our data reveal that variants of the NURF ATP–dependent chromatin remodeling complex that recognize post-translational histone modifications are important regulators of primary spermatocyte differentiation in *Drosophila*.

## Author Summary

Changes in nucleosome dynamics have a profound effect on DNA transactions such as transcription, replication, and repair. Altered chromatin states can be induced by post-translational modification of the histone tails or energy-dependent nucleosome sliding mediated by ATP–dependent chromatin remodeling factors. Here we demonstrate that the *Drosophila* chromatin remodeling factor NURF is regulated by alternative splicing of its large subunit NURF301. We show that three NURF301 isoforms occur. One of these lacks C-terminal protein domains that recognize the post-translational histone modifications H3K4me3 and H4K16Ac and that potentially allow recruitment of NURF to modified histone marks. Using whole genome expression profiling, we identify NURF target genes that require these domains and, hence, recognition of modified histone marks. Our results indicate that NURF complexes that recognize H3K4me3 and H4K16Ac are not essential for correct expression of the majority of NURF gene targets in larvae but are obligatory for NURF function in spermatogenesis. We show that NURF is an important regulator of spermatocyte differentiation in *Drosophila*. We suggest that alternative splicing provides a convenient mechanism to generate functional diversity of ATP–dependent chromatin remodeling complexes and allows the production of remodeling complexes with altered chromatin targeting specificities.

## Introduction

The organization of DNA in nucleosomes has a vital function in regulating whether genetic information can be expressed. By altering nucleosome dynamics, genes can be rendered inaccessible or made available to the transcription machinery. There are a number of mechanisms by which altered chromatin states can be induced. Post-translational modification of the histone tails can change associations between histones and DNA and between neighboring nucleosomes, altering chromatin flexibility and conformation (reviewed in [1]–[3]). A second approach is through the deployment of ATP-dependent chromatin remodeling factors (reviewed in [4],[5]). These multi-subunit protein complexes utilize the energy of ATP hydrolysis to alter the dynamic properties of nucleosomes. Traditionally they are divided into broad families depending on the catalytic subunit utilized, and their mechanism of altering nucleosomes - either nucleosome eviction, sliding or variant histone replacement.

The principal activity of the ISWI family of ATP-dependent chromatin remodeling factors is energy-dependent nucleosome sliding [6]–[8]. The nucleosome remodeling factor (NURF) is one of the founding members of this family. By sliding nucleosomes, NURF can alternatively expose or occlude transcription factor binding sites, activating or repressing transcription. Consistent with this, studies of mutants that lack either the NURF-specific large subunit NURF301, or the catalytic ISWI ATPase subunit have shown that NURF is required for both transcription activation and repression *in vivo* [9]–[12]. Gene targets that require NURF for activation include the homeotic selector genes *engrailed*, *Ultrabithorax*, ecdysone responsive genes and the *roX* non-coding RNA [9],[12],[13],[14]. In contrast, NURF has been shown to regulate *Drosophila* innate immunity by repressing targets of the JAK/STAT pathway [15].

Although NURF is composed of four subunits, only the largest subunit (in *Drosophila* NURF301; in humans bromodomain and PHD finger transcription factor (BPTF)) is specific to NURF [8],[16]. All other subunits are present in a number of other chromatin remodeling complexes. This suggests that targeting of NURF to promoters is likely to be a function of NURF301/BPTF. Consistent with this, NURF301/BPTF contains PHD (plant homeo domain) fingers and a bromodomain, motifs that have the potential to recognize specific histone modifications. The C-terminal PHD finger of BPTF/NURF binds the amino terminal tail of histone H3 trimethylated at lysine position 4 (H3K4me3) and has been shown to recruit NURF to sites of H3K4me3 enrichment in humans [17]. In addition, structural studies of the bromodomain of BPTF predict that it can recognize histone H4 acetylated at lysine position 16 (H4K16Ac) [18]. This suggests that NURF may bind to sites of H4K16Ac, although *in vitro* studies indicate that H4K16Ac may also inhibit catalytic activity of the ISWI subunit of NURF and hence NURF-mediated chromatin remodeling [19],[20].

Alternative splicing presents another mechanism by which the activity of chromatin modifying and remodeling factors may be regulated. Sequence analysis of 160 chromatin-associated proteins has revealed that 28% of these encode alternative splice forms in which domains critical for function are substituted [21]. Consistent with this, Shiekhattar and colleagues [22] have previously reported that function of human NURF can be regulated by the incorporation of a catalytically inactive isoform of the SNF2L subunit. In addition, splice variants of SNF2L that lack nuclear localization signals have also been identified [23]. This raises the possibility that multiple distinct NURF complexes exist with altered functions conferred by the incorporation of splice variants of the major subunits. In this report we examine whether *Drosophila* NURF is subject to alternative splicing. We show that three NURF301 isoforms occur, one of which lacks the PHD fingers and bromodomains that recognize the H3K4me3 and H4K16Ac marks. Using whole genome expression profiling we identify NURF target genes that require these domains and hence recognition of histone tail modifications. Our results indicate that NURF complexes that recognize H3K4me3 and H4K16Ac are not required for correct expression of the majority of NURF gene targets in larvae, but are obligatory for NURF function in spermatogenesis. Our data reveals that alternative splicing of the large specificity subunit NURF301 allows the elaboration of functionally distinct variants of the NURF ATP-dependent chromatin remodeling complex.

## Results

### Naturally occurring isoforms of NURF301

**Figure 1:**
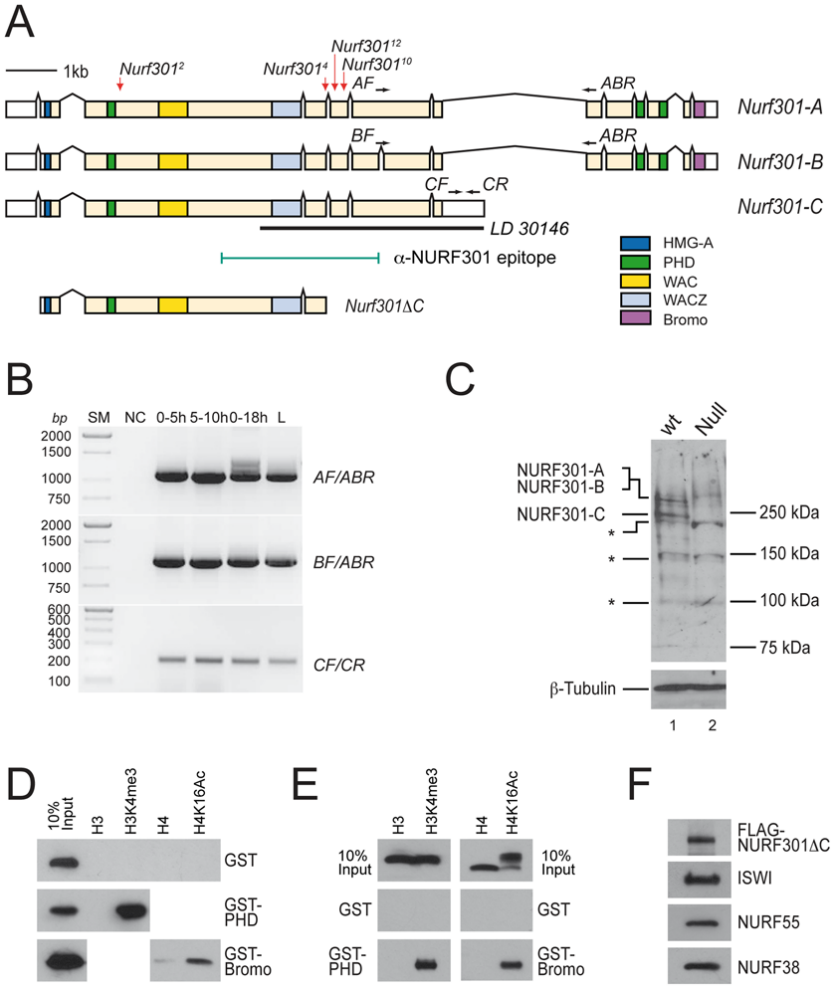
Isoforms of NURF301. (A) *Nurf301* locus indicating three predicted isoforms. The locations of isoform-specific RT-PCR primers and the anti-NURF301 antibody epitope are indicated. The location of the EMS mutations *Nurf301^2^*, *Nurf301^4^*, *Nurf301^10^*, and *Nurf301^12^* are denoted by red arrows above the *Nurf301-A* schematic. The cDNA expressed by these mutations (*Nurf301ΔC*) is indicated beneath the schematic. (B) RT–PCR using isoform-specific primers (Table S1) detects transcripts corresponding to all three isoforms. 0–5 h, 5–10 h, 0–18 h embryo RNA and 3^rd^ instar larval RNA (L) samples were assayed. SM, size marker; NC, no template control. (C) Anti-NURF301 antibodies detect two bands corresponding to NURF301-A/NURF301-B and NURF301-C in wild-type (*wt*) extracts. Both bands are lost in null *Nurf301^2^* mutant (*Null*) extracts. β-Tubulin is used as a loading control. Asterisks indicate cross-reactive protein species detected in all genotypes. (D) Biotinylated histone tail peptides (H3 and H4 tail peptides lacking modifications, H3K4me3 and H4K16Ac (all amino acids 1–21)) were bound to streptavidin agarose beads and incubated with purified GST, GST-PHD and GST-Bromo proteins. H3K4me3 modified peptide pulled-down GST-PHD while H4K16Ac modified peptide pulled-down GST-Bromo. No interaction was observed with GST. (E) Reciprocal pull-down using glutathione-coated beads to pull-down purified GST-fusion proteins and interacting tail peptides. Strong binding between GST-PHD and H3K4me3, and GST-Bromo and H4K16Ac was observed. The bromodomain-H4K16Ac interaction was stronger than that observed in (D), probably due to the proximity of the modification to the bead surface in peptide driven pull-downs. (F) Western blotting indicates that FLAG-tagged truncated NURF301 (FLAG-NURF301ΔC, amino acids 1–1557, corresponding to the protein product encoded by the *Nurf301ΔC* mutation *Nurf301^10^*) co-purifies with ISWI, NURF55 and NURF38.

Inspection of the *Drosophila* genome sequence (Release 5.2 annotation, http://flybase.bio.indiana.edu/) revealed that the locus encoding the NURF specificity subunit (*Nurf301*) potentially generates three isoforms by alternative splicing (Figure 1A). *Nurf301-A* corresponds to the previously described full-length isoform [8]. *Nurf301-B* contains an additional intron that deletes 60 bp from exon 6. Most interestingly, *Nurf301-C* is a truncated isoform that terminates after the 7^th^ exon. This truncation removes the C-terminal PHD fingers and bromodomain that are present in *Nurf301-A* and *Nurf301-B*. These domains are predicted to bind histone modifications, raising the possibility that distinct classes of NURF complexes exist, one built around NURF301-A or NURF301-B that can recognize the H3K4me3 and H4K16Ac histone modifications, and a second founded on NURF301-C that cannot.

As a first step to verify this we confirmed these isoform predictions. RT-PCR using isoform-specific primer sets revealed that all three isoforms were expressed (Figure 1B). No apparent differences in the distribution of expression were observed. Further support was provided by Northern analysis, which detected transcripts corresponding to the *Nurf301-A*, *Nurf301-B* and *Nurf301-C* isoforms (Figure S1A). Additional evidence for the existence of the *Nurf301-C* isoform was provided by the presence of an EST (LD30146) within the Berkeley Drosophila Genome Project collection that corresponds to the 3′ region of *Nurf301-C* (Figure 1A). Moreover, sequence comparison of the *Nurf301* genomic interval flanking exon 7 from *Drosophila melanogaster*, *D. pseudobscura* and *D. mojavensis* reveals nucleotide sequence conservation typical of coding sequence up to the predicted stop codon of *Nurf301-C* in all species (Figure S1C). Finally, western analysis, using an anti-NURF301 antibody that recognizes an epitope shared by all predicted isoforms [15], demonstrated that the transcripts encode *bona fide* protein isoforms. In extracts of third instar larval imaginal discs, protein species migrating at 301 kDa and 240 kDa were detected (Figure 1C, lane 1). These bands correspond with the predicted sizes of the NURF301-A and NURF301-B isoforms, which co-migrate, and the NURF301-C isoform. Neither band was observed in extracts of null *Nurf301* mutants that disrupt all isoforms (Figure 1C, lane 2).

We next verified which histone modifications were bound by the C-terminal PHD finger and bromodomain of full-length NURF301. Single domains were expressed and purified as GST-fusion proteins and tested for the ability to bind either unmodified histone H3 and H4 tail peptides, H3K4me3 tail peptide or H4K16Ac tail peptide. Pull-downs were performed using either the peptides (Figure 1D) or GST-fusion proteins (Figure 1E) as baits. As observed previously [17], the C-terminal PHD finger of NURF301 was able to bind H3K4me3 tail peptide but did not bind unmodified H3 tail peptide (GST-PHD in Figure 1D and 1E). Likewise the bromodomain was able to bind H4K16Ac tail peptide but did not bind unmodified H4 tail peptide (GST-Bromo in Figure 1D and 1E).

Our data indicate that NURF complexes built around full-length NURF301-A or around NURF301-B should have the ability to bind both H3K4me3 and H4K16Ac histone modifications. However, NURF complexes composed of the NURF301-C isoform will lack this binding ability. To confirm whether truncated NURF301 isoforms would be able to assemble a NURF complex, we determined association with the three other subunits of the NURF complex: ISWI, NURF55 and NURF38. Insect cells were co-infected with baculoviruses that express ISWI, NURF55, NURF38 and a FLAG-tagged C-terminally truncated version of NURF301 (FLAG-NURF301ΔC). FLAG-NURF301ΔC was then purified using anti-FLAG beads and association of other NURF subunits assayed by Western blotting. As shown in Figure 1F, ISWI, NURF55 and NURF38 co-purified with the truncated NUR301ΔC isoform. This defines a minimal ISWI-, NURF55- and NURF38-interacting region that is present within all NURF301 isoforms. It has previously been shown that the ISWI subunit alone displays remodeling activity, and that this does not require the presence of the other NURF subunits [24]. Thus, by associating with ISWI, all NURF301 isoforms should nucleate the formation of functional chromatin remodeling complexes. To distinguish these alternative NURF complexes, we have designated them NURF-A, NURF-B and NURF-C, corresponding to the NURF301 isoform utilized. For example, NURF-C indicates the NURF complex constituted from the short PHD finger- and bromodomain-deficient isoform NURF301-C.

### Whole genome expression profiling

To address the function of these complexes, in particular to distinguish functions of the full-length NURF-A and NURF-B complexes from the NURF-C complex, we have made use of a cluster of EMS-induced mutations that truncate *Nurf301* at exon 5. As shown in Figure 1A, these mutations, which we term *Nurf301ΔC* mutations, generate shortened NURF301 isoforms that contain all of the conserved motifs present in NURF301 except the C-terminal PHD fingers and bromodomain. These mutations will ablate function of NURF301-A and NURF301-B while still expressing a protein variant (NURF301ΔC) that is a reasonable model for NURF301-C. As described previously [13], *Nurf301^4^* corresponds to a splice donor mutation that truncates NURF301 after amino acid 1528, while *Nurf301^10^* and *Nurf301^12^* are amino acid to stop mutations that truncate NURF301 at amino acids 1557 and 1532 respectively. Western analysis of extracts of *Nurf301ΔC* mutants confirmed that they encode a truncated, but stable, protein of the expected molecular mass of 180 kDa (Figure S1B). Comparison of these *Nurf301ΔC* mutants (that lack NURF301-A and NURF301-B) with either wild-type animals or a null *Nurf301* mutant (*Nurf301^2^*) that disrupts all NURF301 isoforms, allows the reasonable discrimination of the relative requirements of the full-length (NURF-A and NURF-B) and NURF-C complexes.

**Figure 2:**
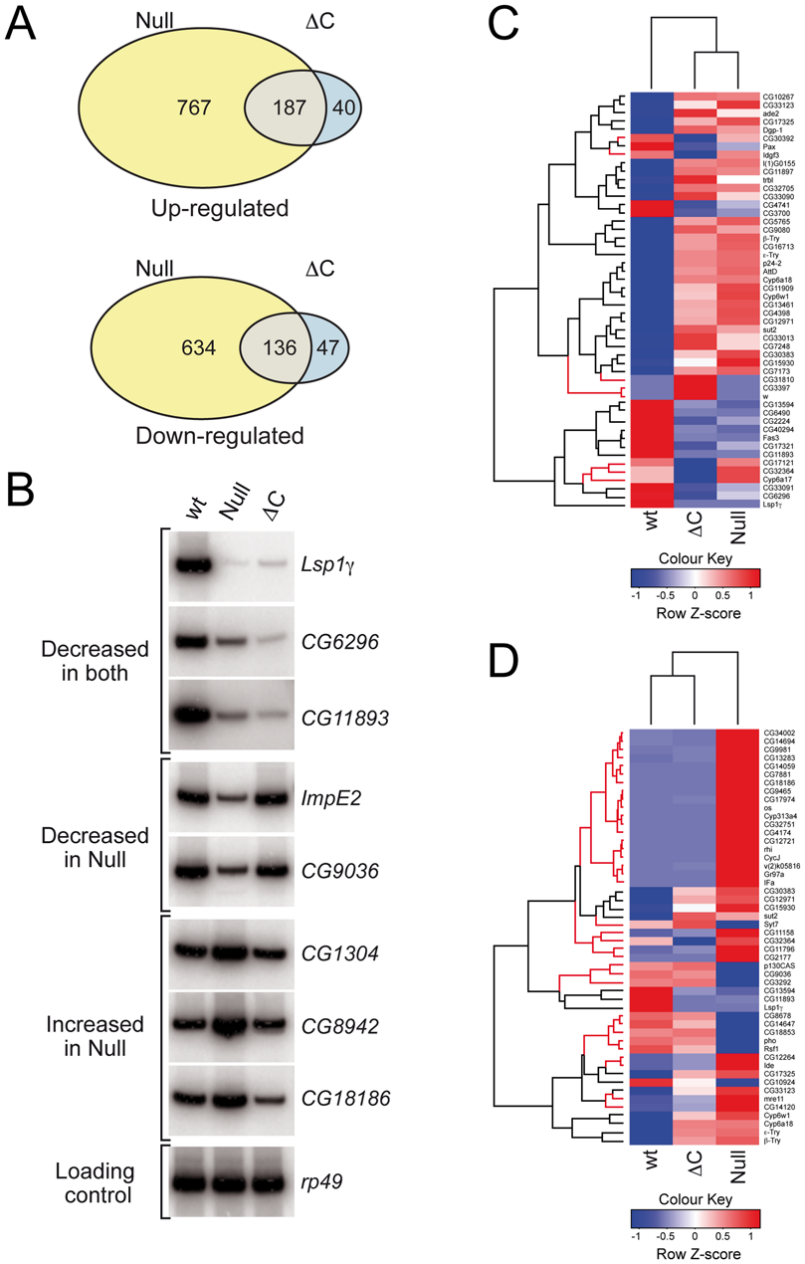
A subset of NURF targets requires the PHD fingers and bromodomain. (A) Venn diagrams indicating overlap in transcriptomes of null *Nurf301* and *Nurf301ΔC* mutants. Yellow indicates genes altered in null *Nurf301* mutants, blue genes changed in *Nurf301ΔC*, and grey genes regulated in common. (B) Abundance of selected transcripts was analyzed in mRNA isolated from whole wild type, null *Nurf301* and *Nurf301ΔC* mutant third instar larvae. Transcript abundance is normalized to *Ribosomal protein L32* (*rp49*) as a loading control. (C, D) Heatdiagram of the 50 genes with the most statistically-significant divergence in expression relative to wild-type for (C) *Nurf301ΔC* and (D) null *Nurf301* mutants. Red branches denote differences in expression between *Nurf301ΔC* and null *Nurf301* datasets.

As a first step, we used whole genome expression profiling to identify gene targets that show altered expression in either the null *Nurf301* or *Nurf301ΔC* mutants relative to wild-type. mRNA was isolated from third instar larvae of either *Nurf301* mutant strain and from the parental isogenic strain in which these mutants were generated. Samples were labeled and hybridized to Affymetrix *Drosophila* Genome 2.0 arrays. We then used the *limma* package [25] to determine genes with a statistically significant (*P<0.01*) change in expression relative to the wild-type control in either *Nurf301* mutant. As shown in Figure 2A, Only 20% (187) of the genes that were up-regulated, and 21% (136) of the genes that were down-regulated in null *Nurf301* mutants were similarly changed in *Nurf301ΔC* mutants. This indicates that NURF-C is sufficient for expression of the majority of NURF target genes and that recognition of H3K4me3 and H4K16Ac is only obligatory for a subset of these in larvae.

RT-PCR for selected transcripts with the most statistically-significant changes in expression confirmed the microarray results. As shown in Figure 2B, NURF targets that required full-length NURF301 included *Lsp1γ*, *CG6296* and *CG11893*. Expression of these genes was reduced in both mutants (Figure 2B). Interestingly, real-time PCR analysis of transcript abundance (Figure S2) indicated that levels of *CG6296* and *CG11893* were reduced more in *Nurf301ΔC* mutants than null *Nurf301* mutants, raising the possibility of antagonistic functions of full-length and NURF-C complexes. In contrast, expression of *ImpE2* and *CG9036* was unaffected in *Nurf301ΔC* mutants (Figure 2B), indicating that these are targets of NURF-C alone. Similarly, up-regulation of *CG1304*, *CG8942* and *CG18186* only occurred in null *Nurf301* mutants and was not observed in *Nurf301ΔC* mutants, suggesting that NURF-C is sufficient for repression of these.

Further evidence that NURF-C is sufficient, and that recognition of H3K4me3 and H4K16Ac is not obligatory for correct expression of the majority of NURF target genes, was provided hierarchical clustering of the microarray data. The top 50 genes that showed the most statistically-significant difference in expression relative to wild-type (lowest adjusted *P*-value) in either the *Nurf301ΔC* (Figure 2C) or null *Nurf301* mutants (Figure 2D) were selected. Genes and samples were then clustered according to expression in all three genotypes. This revealed that only 16% of genes that show altered expression when all NURF301 isoforms are removed (null mutants) were similarly affected when NURF-C is present (*Nurf301ΔC* mutants). Conversely, most genes with altered expression in *Nurf301ΔC* mutants were similarly changed in null *Nurf301* mutants.

### *Nurf301ΔC* mutants do not up-regulate the JAK/STAT pathway

Previously we reported that null *Nurf301* mutants exhibit de-regulated immune responses and develop inflammatory (melanotic) tumours [10],[15]. In these studies it was shown that NURF acts as a co-repressor of a subset of JAK/STAT target genes [15]. Therefore we investigated whether repression of the JAK/STAT pathway requires interpretation of histone modifications by full-length NURF301. First we determined whether any of the genes we previously showed to be regulated by NURF and the JAK/STAT pathway [15] were also affected in *Nurf301ΔC* mutants. Analysis of *Nurf301ΔC* microarray data revealed that just 14 out of the 119 NURF and JAK/STAT target genes identified before show any significant alterations in expression (change P value<0.05 and fold change +/−2.5), indicating that NURF301-C is sufficient for repression of the majority of JAK/STAT targets.

**Figure 3:**
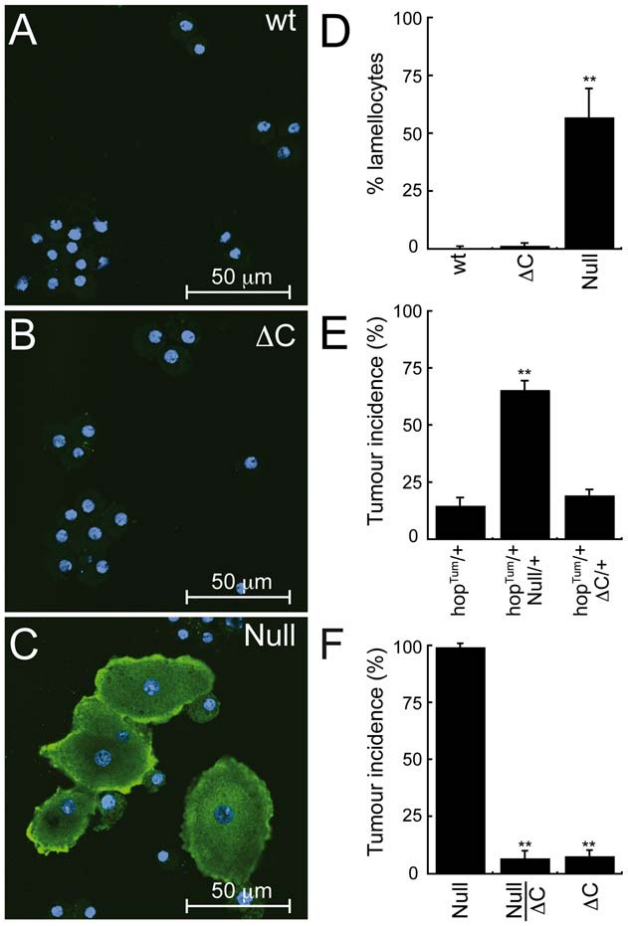
Innate immune responses do not require interpretation of histone modifications by NURF. Hemocytes from (A) wild-type, (B) *Nurf301ΔC*, and (C) null *Nurf301* mutant third instar larvae were immunostained with the lamellocyte-specific antibody MAb L1b. MAb L1b staining is revealed in green, DAPI-stained nuclei are shown in blue. Increased lamellocyte number is only observed in (C) null *Nurf301* mutant third instar larvae. (D) Lamellocyte frequency was determined as the ratio of MAb L1-positive cells relative to total hemocyte number (marked by DAPI staining) in a field. Values are the mean+/−s.d. of 5 determinations for each genotype. Double asterisk indicates values significantly different from wild-type (Student's *t*-test, *P*<0.01). (E) The incidence of melanotic tumors is increased in adult female flies simultaneously heterozygous for *hop^Tum^* (gain-of-function JAK) and null *Nurf301* mutations. No increase in melanotic tumors incidence is recorded in flies simultaneously heterozygous for *hop^Tum^* and *Nurf301ΔC* mutations (*Nurf301^4^*). Values are the mean+/−s.d. of 5 determinations for each genotype (each determination a minimum of 30 flies). Double asterisk indicates values significantly different from *hop^Tum^*/+ control flies (Student's *t*-test, *P*<0.01). (F) Melanotic tumor incidence in homozygous null *Nurf301* mutant third instar larvae approaches 100%. In contrast, null *Nurf301/Nurf301ΔC* (*Nurf301^2^/Nurf301^4^*) or *Nurf301ΔC* (*Nurf301^4^*) homozygous mutant larvae show significantly reduced tumour incidence. Values are the mean+/−s.d. of 3 determinations for each genotype (each determination minimum of 30 larvae). Double asterisk indicates values significantly different from null *Nurf301* homozygous larvae (Student's *t*-test, *P*<0.01).

The failure to induce the JAK/STAT pathway in *Nurf301ΔC* mutants was confirmed by determining lamellocyte numbers. In null *Nurf301* mutants over-activation of the JAK/STAT pathway leads to the differentiation of large numbers lamellocytes, usually a rare hemocyte cell type (compare Figure 3A and 3C), with lamellocyte frequency increasing from 0.44% to 57% (Figure 3D). However, in *Nurf301ΔC* mutants (Figure 3B) lamellocyte numbers did not increase, exhibiting an average lamellocyte frequency of 1.4%, not statistically significant different from wild-type lamellocyte frequencies.

As additional proof that repression of the JAK/STAT pathway does not require the NURF-A and NURF-B complexes, we examined whether *Nurf301ΔC* mutations interact genetically with the JAK/STAT pathway. A gain-of-function mutation in the *Drosophila* JAK (*hop^Tum^*) induces temperature dependant constitutive activation of the JAK/STAT pathway causing the formation of melanotic tumors [26],[27]. As we have reported previously [10], null *Nurf301* mutants are genetic enhancers of *hop^Tum^*, increasing tumor incidence from 14.7% to 65.3%. In contrast *Nurf301ΔC* mutants were not enhancers of the *hop^Tum^* mutation (Figure 3E).

Finally, we compared melanotic tumour frequencies in all *Nurf301* mutant larvae. The development of inflammatory melanotic tumours is the ultimate morphological outcome of constitutive activation of the JAK/STAT pathway. As shown in Figure 3F, almost 100% of null *Nurf301* mutants developed melanotic tumours. Conversely, only 6.6% of null *Nurf301/Nurf301ΔC* transheterozygous animals or 7.5% of *Nurf301ΔC* homozygous mutant larvae developed tumours. This is consistent with the reduced activation of immune targets in *Nurf301ΔC* observed during microarray analysis and the failure to increase lamellocyte numbers or activate the JAK/STAT pathway significantly. Taken together, these results indicate that repression of the JAK/STAT pathway by NURF does not require interpretation of histone modifications by the NURF-A and NURF-B complexes.

### The C-terminus of NURF301 is required for spermatogenesis

Whole genome expression profiling data from larval stages indicated that correct interpretation of histone modification marks is required for only a subset of NURF function at larval stages. Indeed, unlike null *Nurf301* mutants, *Nurf301ΔC* mutants are viable until adult stages. Interestingly, however, *Nurf301ΔC* mutants are both male and female sterile suggesting that full length NURF301 is obligatory for gametogenesis. To identify the defect in spermatogenesis, testes were dissected from wild-type and *Nurf301ΔC* mutant males and examined by phase contrast microscopy. As reviewed in Fuller (1993) [28], gonial cells arise from stem cells located at the tip of the testis surrounding a specialized structure called the hub. Gonial cells undergo four transit-amplifying mitotic divisions to yield 16 primary spermatocytes. These cells then enter an extended G2 phase during which cell volume increases and transcription of genes required for meiosis and post-meiotic spermatid differentiation occurs. After two meiotic divisions spermatid differentiation occurs with the eventual production of motile sperm.

**Figure 4:**
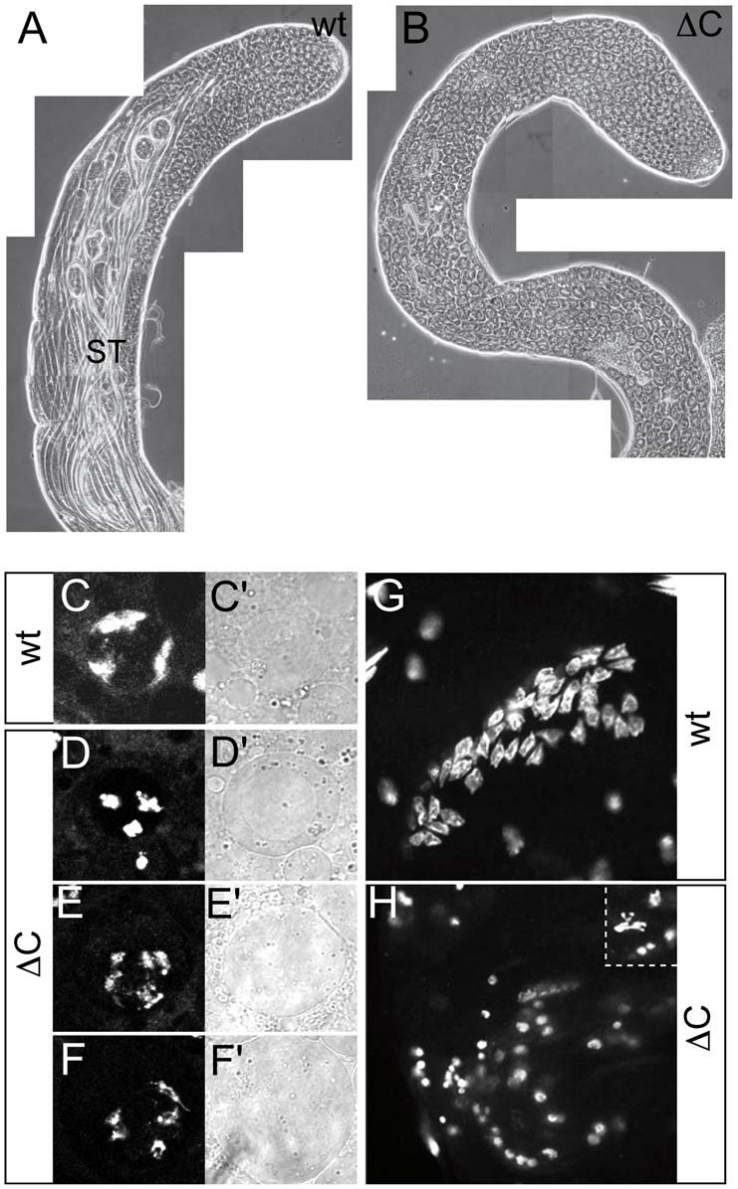
Primary spermatocyte arrest phenotype of *Nurf301ΔC* mutants. Phase microscopy of live testes dissected from (A) wild type, and (B) *Nurf301ΔC* (*Nurf301^4^*) mutant males indicates that mature sperm are not formed in *Nurf301ΔC* mutant testes. Instead characteristic large round primary spermatocytes accumulate. Mature sperm tails in wild type are labeled (ST) for comparison. (C) Hoechst 33258 staining of wild type primary spermatocytes reveals the typical peripheral sectored organization of the principal chromosome bivalents. (D–F) Bivalents in *Nurf301ΔC* mutant primary spermatocytes show aberrant organization, appearing more compacted and punctate at initial stages while at later stages, fragmentation and separation of the bivalents occurs. (C′–F′) Panels show phase contrast images of the corresponding primary spermatocytes in (C–F). In rare instances, *Nurf301ΔC* mutant primary spermatocytes enter meiosis and attempt to form spermatids. However, unlike the regular arrays of spermatid nuclei (64 per cyst) seen in wild-type testes (G), only highly fragmented and condensed DNA staining can be observed in *Nurf301ΔC* mutants (H). Inset in panel (H) shows high magnification of fragmented nuclei in *Nurf301ΔC* mutants.

As shown in Figure 4A, bundles of differentiated sperm were easily resolved by phase microscopy in live wild-type testes. However, in *Nurf301ΔC* mutant testes, no sperm bundles were observed indicating that mature sperm were not formed (Figure 4B). Instead, large numbers of primary spermatocytes accumulated, suggesting that there is a defect in further differentiation and meiotic division of primary spermatocytes. This was confirmed by staining of testes with the DNA dye Hoechst 33342 to resolve nuclear morphology. In wild-type primary spermatocytes, the chromosome bivalents formed three distinct domains located near the nuclear periphery (Figure 4C). In *Nurf301ΔC* mutants the bivalents formed three distinct territories but the chromosomes appeared more compact (Figure 4D) and progressive separation and fragmentation of the bivalents occurred (Figure 4E and 4F). In rare cases primary spermatocytes in *Nurf301ΔC* mutants attempted the two meiotic divisions that generate spermatids. However, as shown in Figure 4H nuclei were highly disrupted and fragmented compared with wild-type spermatid nuclei (Figure 4G).

### Defects in primary spermatocyte differentiation in *Nurf301ΔC* mutants

The defects that occur in primary spermatocyte differentiation in *Nurf301ΔC* mutants were strikingly similar to those that occur in a class of male sterile mutants known as meiotic arrest mutants. Mutants in the genes *spermatocyte arrest* (*sa*), *cannonball* (*can*), *always early* (*aly*) and *meiosis I arrest* (*mia*) result in the accumulation of mature primary spermatocytes and the absence of post-meiotic phases [29]. These gene products act to coordinate spermatid differentiation and meiotic cell cycle, with all four controlling transcription of spermatid differentiation genes in primary spermatocytes and *aly* also required for transcription of *cyclin B* and thus meiotic entry [30]. The similarity of the testes phenotypes of the *Nurf301ΔC* mutants and the meiotic arrest mutants led us to determine whether full-length NURF301 was also required for expression of spermatid differentiation genes and meiotic cell cycle regulators.

**Figure 5:**
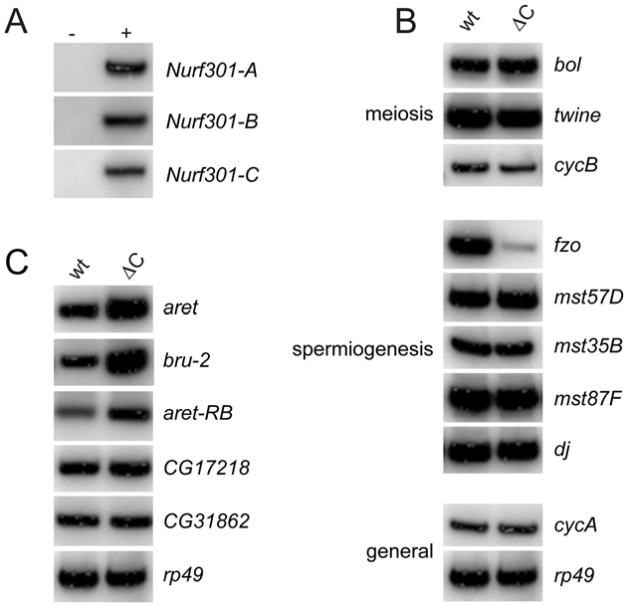
NURF301 isoform expression and transcriptional targets of NURF in testes. (A) RT–PCR using isoform-specific primer sets confirms that all three *Nurf301* isoforms are expressed in testes. (−) and (+) indicates absence of presence of cDNA template in otherwise identical PCR reactions. (B) RT-PCR was performed on mRNA isolated from wild-type and *Nurf301ΔC* (*Nurf301^4^*) mutant testes. Primers corresponding to known targets of tTAFs define *fzo* as a NURF testis transcriptional target. Transcripts analyzed included meiotic regulators *bol*, *twine* and *cyclin B*, and genes required for spermiogenesis *fzo*, *mst57D*, *mst35B*, *mst87F* and *don juan*. *cyclin A* (*cycA*) and *rp49* transcripts are general transcripts that served as loading controls. (C) Transcript levels of both *aret* and *bru-2* are elevated in *Nurf301ΔC* testes providing a mechanism for repression of Cyclin B accumulation. Levels of the intervening transcript *CG31862* and flanking transcript *CG17218* are unaffected when compared relative to the loading control *rp49*.

As shown in Figure 5A, we first validated that all three NURF isoforms were expressed in testes. RT-PCR confirmed that the full-length *Nurf301-A* and *Nurf301-B* transcripts were expressed in testes, as was the *Nurf301-C* isoform that lacks the bromodomain and PHD fingers. We next tested whether transcription of meiotic cell cycle regulators was disrupted in the *Nurf301ΔC* mutants. Unlike *aly* mutants, in which transcription of *cyclin B* (*cycB*) is lost, *cycB* transcription in *Nurf301ΔC* mutants was unchanged relative to wild-type testes, as was transcription of *twine* and *boule (bol)* (Figure 5B). We then assessed whether transcription of any of the spermatid differentiation genes known to be affected in *can*, *sa*, and *mia* mutants required NURF for expression. As shown in Figure 5B, of the genes examined, only expression of *fuzzy onions* (*fzo*) was reduced in *Nurf301ΔC* mutant testes when compared with wild-type testes. Moreover unlike *can*, *sa*, and *mia* mutants in which protein levels of Bol are reduced, in *Nurf301ΔC* mutant testes Bol levels were unaffected (Figure S3C). These results were also confirmed by real-time analysis of transcript abundance (Figure S4).

### NURF represses the translational inhibitor Bruno to control cyclin B expression

Failure to express *fzo* suggests that NURF-A is required to regulate expression of a subset of spermatid differentiation genes. However loss of *fzo* expression does not account for the meiotic arrest phenotype seen in *Nurf301ΔC* mutants, as *fzo* mutants show no defects in meiosis, only exhibiting a defect at later stages of spermiogenesis [31]. We thus examined if protein expression of any of the meiotic cell cycle regulators was affected in *Nurf301ΔC* mutants. Interestingly staining with antibodies against Cyclin B, revealed that levels of Cyclin B protein were decreased in primary spermatocytes of *Nurf301ΔC* mutants compared with wild-type primary spermatocytes (Figure S3). The failure to express Cyclin B protein is predicted to arrest spermatocytes at the G2-M transition.

As *cycB* transcript levels are unaffected in *Nurf301ΔC* mutants (Figure 5B), regulation is likely to occur through control of protein stability or protein translation. Sugimura and Lilly (2006) [32] have reported that Cyclin B protein levels can be regulated in female meiosis by the translational inhibitor Bruno (Bru), which is encoded by the gene *arrest* (*aret*). In *Drosophila* three Bruno family members occur. *aret* and *bruno-2* (*bru-2*) are located adjacent to each other on chromosome 2 L, while *bruno-3* (*bru-3*) is located on chromosome 3 L (Figure S5). All are RRM (RNA-recognition motif) containing proteins, with Bru and Bruno-2 (Bru-2) being most similar [33],[34]. Interestingly, we observed previously by whole genome expression profiling that levels of Bru family members were up-regulated in *Nurf301* mutant tissues (S.Y.K and P.B., unpublished data). We therefore examined whether levels of Bru or Bru-2 were up-regulated in *Nurf301ΔC* mutants testes. Transcript levels of *bru-3* were unaffected in *Nurf301ΔC* mutant testes (data not shown). However, as shown in Figure 5C, both *aret* (Bru) and *bru-2* transcript levels were elevated in mutant testes. Elevation of transcript levels was specific to the Bruno family members and not a general feature of this chromosome interval as levels of the flanking gene (*CG17218*) and the intervening gene (*CG31862*) were unchanged (Figure 5C). In adults, normally four *aret* transcripts can be detected: three female-specific transcripts and a single male specific transcript [35]. Using primers that detect only the male-specific Bru transcript (*aret-RB*) we observed that levels of the male-specific transcript were elevated in *Nurf301ΔC* mutant testes. We conclude that the block to meiotic entry in *Nurf301ΔC* mutant testes is mediated by translational repression of Cyclin B expression caused by up-regulation of the Bru translational inhibitors.

### NURF–binding overlaps the histone modifications H3K4me3 and H4K16Ac

**Figure 6:**
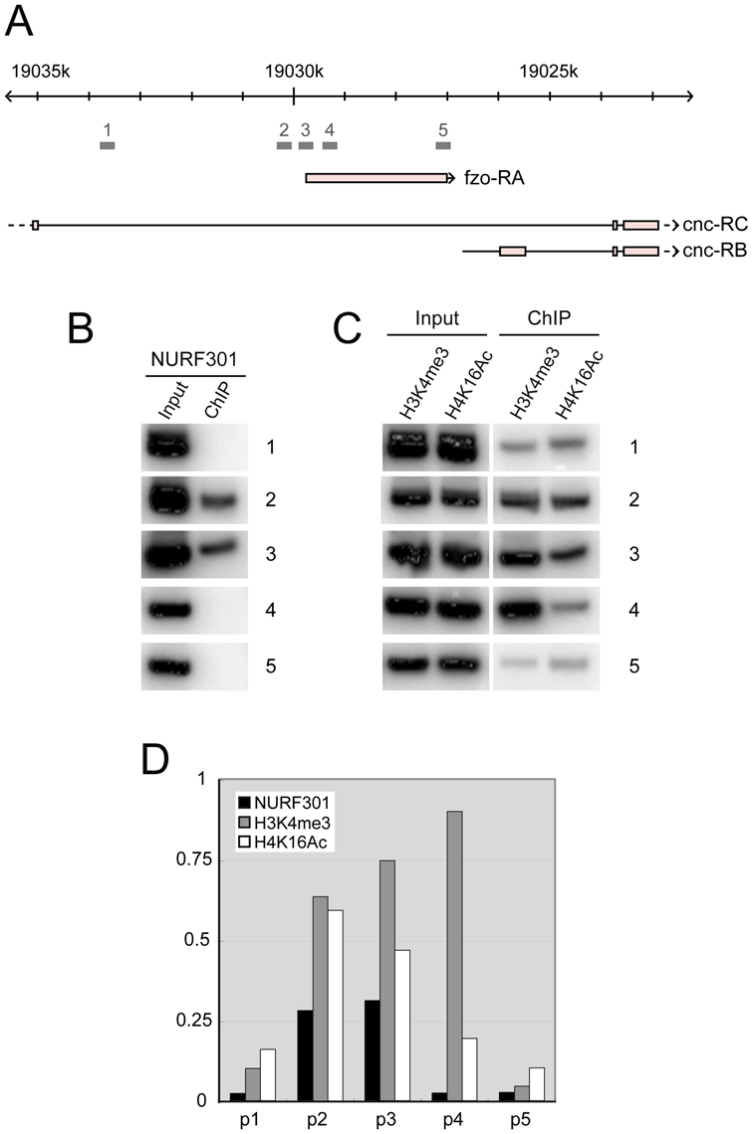
NURF binds *fzo* and overlaps the H3K4me3 and H4K16Ac marks. (A) Structure of the *fzo* locus indicating primer pairs used in ChIP analysis. *fzo* is nested within the *cap-n-collar* (*cnc*) gene, located within the 5^th^ intron of the *cnc-RC* transcript. (B) ChIP using anti-NURF301 antibodies indicates that NURF localizes to the promoter and transcriptional start site of *fzo* (primer sets 2 and 3). No binding is detected in the far upstream region (primer set 1), coding region (primer set 4) or 3′ UTR (primer set 5). PCR signals from input and ChIP are included for comparison. (C) ChIP using anti-H3K4me3 antibodies (H3K4me3) or anti-H4K16Ac antibodies (H4K16Ac) indicates that NURF-binding overlaps regions of H3K4me3 and H4K16Ac accumulation. PCR signals from input and ChIP are included for comparison. (D) Plot of ChIP signal intensities normalized to input signals for anti-NURF301, anti-H3K4me3 and anti-H4K16Ac ChIPs, indicating overlap of NURF binding and histone modifications.

Next we analyzed whether regulation of *fzo* by NURF is direct and whether NURF binding coincides with histone modifications. We assayed NURF binding by chromatin immunoprecipitation (ChIP) using antibodies against NURF301 and were able to detect binding of NURF to *fzo* in testes (Figure 6). NURF-binding overlapped the promoter and transcription start site of *fzo* (Figure 6B, primer sets 2 and 3) but was not detected in the far upstream region (primer set 1), coding region (primer set 4) or 3′UTR (primer set 5). As shown in Figure 6C, NURF binding overlapped histone modifications bound by the PHD finger (H3K4me3) and bromodomain (H4K16Ac) of NURF301. We observed that H3K4me3 is detected in the vicinity of the transcription start site (Figure 6C, primer sets 2 and 3) and peaks downstream of the start site (primer set 4). Only low levels of H3K4me3 were detected in the far upstream region (primer set 1) or 3′UTR (primer set 5). H4K16Ac distribution showed a similar profile, however the peak in this modification occurred at the transcription start site (Figure 6C, primer sets 2 and 3) and then declined in the body of the gene (primer sets, 4 and 5). In Figure 6D ChIP signal intensities for NURF, H3K4me3 and H4K16Ac are plotted relative to the corresponding input signals. This data confirmed that NURF-binding overlaps regions accumulating the H3K4me3 and H4K16Ac modifications.

### NURF distribution during primary spermatocyte development

**Figure 7:**
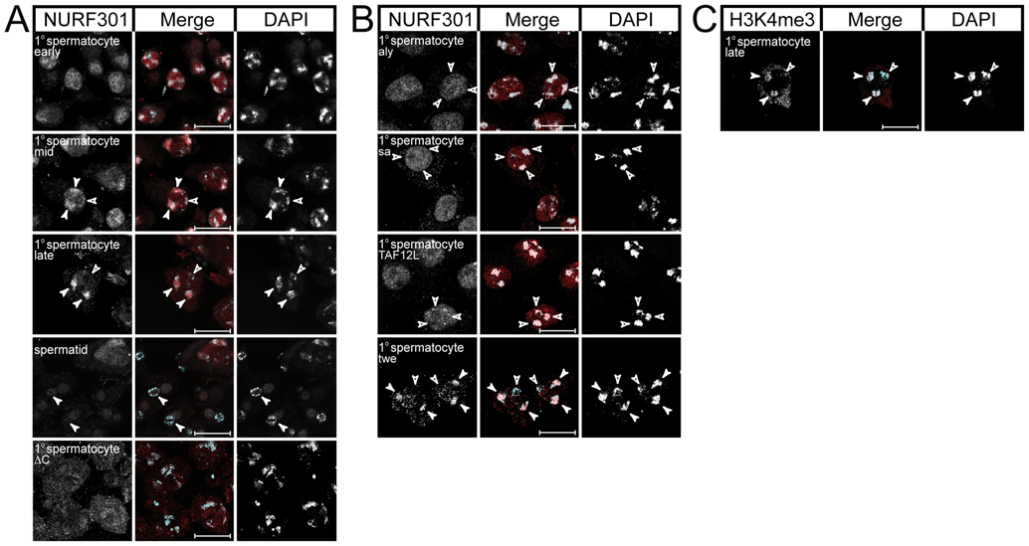
NURF localization in primary spermatocytes. (A) Antibody staining using anti-NURF301 antibodies reveals that NURF is enriched at two of the three bivalents in late primary spermatocytes. In early stage primary spermatocytes NURF is homogenously distributed throughout nuclei. No NURF staining is detected in spermatids. In *Nurf301ΔC* mutant primary spermatocytes no enrichment at bivalents is observed. (B) NURF enrichment on bivalents in mature primary spermatocytes requires function of the meiotic arrest genes *aly*, *sa* and *TAF12L*. NURF staining is unaffected in *twe* mutant primary spermatocytes. (C) H3K4me3, like NURF, is also enriched on two of the three bivalents in late primary spermatocytes. In Merge panels anti-NURF301 and anti-H3K4me3 staining are shown in red, DAPI staining is revealed in blue. Closed arrowheads denote NURF301-staining and H3K4me3-staining bivalents, open arrowheads denote unstained bivalents.

Finally we examined distribution of NURF301 in testes by immunostaining using antibodies that recognize all NURF301 isoforms. As shown in Figure 7, nuclear NURF301 could be detected in spermatocytes, but interestingly distribution within the nucleus was dynamic. In early stage primary spermatocytes, NURF301 staining could be detected throughout the nucleus (Figure 7A). However, during maturation of the primary spermatocytes NURF301 began to accumulate preferentially on the bivalents (Figure 7A, mid) until finally NURF301 was exclusively detected on the bivalents (Figure 7A, late). Interestingly, NURF301 was only enriched on two of the three bivalents (closed arrowheads in Figure 7A). The three bivalents correspond to the second, third, and X and Y chromosomes respectively. The paired second and third chromosomes appear larger and denser than the sex chromatin that surrounds the nucleolus and can be distinguished from these [36]. As such, NURF301 staining can be localized to the second and third chromosome bivalents. Upon completion of meiosis, nuclear levels of NURF301 decline substantially and cannot be detected in nuclei of round spermatids (Figure 7A). *Nurf301ΔC* mutants, which lack the C-terminal PHD fingers and bromodomain, do not show appreciable enrichment of NURF301 on the bivalents (Figure 7A, ΔC). Instead, NURF301 staining is distributed throughout the nuclei of primary spermatocytes suggesting that the C-terminal PHD fingers and bromodomain is required for NURF recruitment to chromatin in spermatocytes. In support of this, antibody staining using anti-H3K4me3 antibodies reveals that H3K4me3 is also selectively enriched on two of the three bivalents, like NURF (Figure 7C).

To establish what other factors play a role in recruitment of NURF to bivalents we examined NURF301 staining in late primary spermatocytes from *aly* mutants, which disrupt the Myb-MuvB/dREAM repressor complex tMAC [37],[38], and from mutants that lack the tTAFs Sa and TAF12L. Loss of both tMAC and tTAFs disrupted NURF301 staining. Although NURF301 was detected in the nuclei of early primary spermatocytes (data not shown), NURF301 failed to localize to the bivalents in mature primary spermatocytes of *aly*, *sa* or *TAF12L* mutants (Figure 7B). As tMAC and tTAFs are required for transcription of genes required both for spermatocyte differentiation as well as meiotic control we examined whether NURF301 staining was disrupted in mutants that block meiotic entry. Twine (Twe) is a germline-specific CDC25 that is required for entry of spermatocytes into meiosis. In *twe* mutants that fail to enter meiosis, NURF301 staining was unaffected (Figure 7B). This suggests that NURF localization requires function of tMAC and tTAFs and that disrupted NURF staining is not an indirect consequence of a meiotic block.

## Discussion

Alternatively splicing represents a convenient mechanism for generating diversity within proteins and protein complexes. In this report we provide evidence that alternative splicing of the large NURF301 subunit of NURF generates functionally distinct chromatin remodeling complexes. We identify two classes of NURF complexes. The first comprises NURF variants composed of full-length NURF301 that have the ability to target the H3K4me3 and H4K16Ac histone modifications. The second consists of NURF complexes composed of a truncated isoform of NURF301 (NURF301-C) that lack this ability. Functional characterization of these variant complexes has allowed the identification of distinct subsets of target genes. We show that NURF complexes that recognize the H3K4me3 and H4K16Ac histone tail modifications are not required for correct expression of the majority of NURF targets in larvae but are obligatory for NURF function in spermatogenesis.

The regulation of *Drosophila* NURF function by alternative splicing of the NURF301 specificity subunit may reflect a general phenomenon that will influence subunits and function of most chromatin remodeling enzymes. This is consistent with analyses of sequence databases that show that 28% of chromatin modifying proteins potentially encode alternative splice forms in which domains critical for function are substituted [21]. A good example of this is SNF2L, the catalytic subunit of human NURF, which generates an inactive isoform by alternative splicing [22],[23]. Moreover, similar truncated NURF301 isoforms are also predicted to occur in other species. For example Andersen and Horwitz (2006) [39] have shown that the homologous *C. elegans nurf-1* locus encodes a variant NURF-1A that lacks the PHD fingers and bromodomain. In addition gene predictions for human BPTF (Swiss Institute of Bioinformatics) and mouse BPTF (UCSC gene predictions) indicate the existence of similar shortened isoforms. We propose that these isoforms do not generate dominant-negative, variant NURF complexes like those produced by alternative splicing of SNF2L [22]. Rather functional NURF remodeling complexes are formed but these possess altered targeting specificity for histone post-translational modifications.

Our data indicate that short NURF301 isoforms are co-expressed with full-length NURF301 in all tissues assayed, and can form a complex with all other NURF subunits. To date we have not uncovered evidence of tissue-specific or exclusive expression of any of the NURF301 isoforms suggesting that these variant NURF complexes co-exist in cells. Thus, functional distinction between these complexes does not seem to be achieved by altering tissue distribution, but rather by regulating their ability to be recruited to target promoters by changing their modified histone-binding properties. Our microarray analysis of null and truncating *Nurf301ΔC* mutants indicates that, for the majority of target promoters in larvae, full-length NURF isoforms are not obligatory for expression. However we do identify a subset of genes that require full-length NURF301 and a number of cases in which the variant NURF complexes appear to have antagonistic functions. For example, we have shown that expression levels of *CG11893* and *CG6296* are reduced by a greater margin in *Nurf301ΔC* than null *Nurf301* mutants, raising the possibility that NURF-C isoforms antagonize function of full-length NURF complexes.

The fact that *Nurf301ΔC* mutants affect expression of only a subset of NURF target genes in larvae is consistent with our previous analyses in which we showed that *Nurf301ΔC* mutants show proper expression of ecdysone target genes [13]. In addition, although null *Nurf301* mutants do not express homeotic genes [9],[10] *Nurf301ΔC* mutants do not show major developmental abnormalities indicating that Hox gene expression is for the most part unaffected. This contrasts with experiments in *Xenopus* that have shown dramatic axial patterning defects as a consequence of loss of H3K4me3 recognition by NURF [17]. One explanation for these differences may be that redundant stabilizing interactions exist on *Drosophila* Hox promoters that reduce the dependency on H3K4me3 for NURF binding to these promoters. This would be consistent with previous data showing that NURF can be recruited to target promoters through interaction with a number of transcription factors, for example the GAGA factor, the ecdysone receptor (EcR) and the *Drosophila* Bcl6 homologue [8],[13],[15]. It seems feasible that, on the majority of NURF targets, interactions with transcription factors may be sufficient for NURF recruitment and activity.

Of the subset of NURF targets that do show altered expression in *Nurf301ΔC* microarrays, approximately equal numbers are up-regulated (227) and down-regulated (183). While some of these may be indirect targets of NURF these data suggest that the C-terminal PHD fingers and Bromodomain may also be required for repression by NURF at some genes. This is confirmed in testes where expression of *aret* and *bru-2* are both up-regulated in *Nurf301ΔC* mutants. There is some evidence that H3K4me3 and H4K16Ac are required for gene repression as binding of H3K4me3 by the PHD finger of ING2 has been shown to be required for transcriptional repression of cyclin D1 [40], and H4K16Ac is required for rDNA silencing by NoRC [41]. However, it is worth noting that the C-terminal region of NURF301 contains a second PHD finger in addition to the distal PHD finger that binds H3K4me3. This PHD finger has all the conserved hydrophobic residues in particular W32 that constitute the aromatic cage shown to be critical for methyl lysine recognition [18], suggesting that it may also bind methylated lysine residues. Experiments are underway to determine the binding specificity of this PHD finger, but it is tempting to speculate that it binds methylated histone lysine residues involved in gene repression. We are currently examining the genome-wide distribution of NURF complexes by ChIP-Sequencing methodologies. Results of these experiments will allow us to determine whether genes derepressed in *Nurf301ΔC* mutants are direct targets of NURF and allow the overlap of NURF-binding with histone marks associated with gene repression to be determined.

Finally, our data indicates that NURF has a key role in *Drosophila* spermatogenesis. We have shown that testis development in *Nurf301ΔC* mutants arrests at the primary spermatocyte stage. We demonstrate that expression of the spermatid differentiation gene *fzo* is lost in *Nurf301ΔC* mutants and that NURF-binding at *fzo* overlaps accumulation of H3K4me3 and H4K16Ac marks. Taken together these data suggests that interpretation of these histone modifications is required for NURF function at the *fzo* promoter. It is important to stress though that these results are correlative. Direct causal requirement for NURF recruitment on H3K4me3 and H4K16Ac would need to be confirmed by loss of NURF under conditions in which these marks are ablated. However, this is hampered by availability of suitable genetic backgrounds in which these modifications are completely removed. Unfortunately, Mof the principal H4K16 acetylase is male lethal as it is required for X-chromosome dosage compensation in males [42]. Moreover, *Trithorax* mutants, which survive to adult stages and have been used to examine *fzo* transcription [43], are not null alleles and do not completely remove H3K4me3 deposition.

The defects that occur in primary spermatocyte differentiation in *Nurf301ΔC* mutants are strikingly similar to those that occur in meiotic arrest mutants [30]. These include the testis-specific TAFs (tTAFs) *nht* (dTAF4), *can* (dTAF5), *mia* (dTAF6), *sa* (dTAF8) and *rye* (dTAF12) [44]. tTAF binding has been shown to be correlated with H3K4me3 accumulation on *fzo*, one of the NURF targets identified in this study, and leads to the displacement of the Polycomb repression complex [43]. It is possible that NURF is the link that integrates the H3K4me3 signal to displace Polycomb. However, meiotic arrest phenotypes are also observed in mutants that lack components of the testis-expressed Myb-MuvB/dREAM repressor complex tMAC [37],[38]. tMAC has been suggested to interact with a testis-specific TFIID composed of tTAFs, in turn regulating transcription in primary spermatocytes [37]. Importantly, NURF is a sub-stoichiometric component of the Myb-MuvB/dREAM complex [45]. Data from *C. elegans* indicates that NURF antagonizes Myb-MuvB/dREAM [39]. This suggests interaction with tMAC may be an alternative route by which NURF regulates transcription in primary spermatocytes. In the future, determining the interactions between NURF, tMAC and tTAFs will help reveal the mechanisms by which these factors regulate transcription in primary spermatocytes.

## Methods

### Genetics and *Drosophila* strains

Flies were raised at 25°C. Selection of *Nurf301^2^*, *Nurf301^4^* and *Nurf301^12^* mutants is described in [10]. *aly^1^*, *sa^1^*, *twe^1^* and *Taf12L^KG00946^* strains were obtained from the Bloomington Drosophila Stock Center.

### Whole genome expression analysis

*w^1118^*, *Nurf301^2^*, and *Nurf301^4^* wandering third instar larvae were staged using the blue-gut method [46]. mRNA was isolated by magnetic selection using µMacs columns according to manufacturer's instructions (Miltenyi Biotec, Auburn, CA). Whole genome expression profiling was performed using Affymetrix Drosophila Genome 2.0 arrays as described in [13]. Statistical analysis was carried out using R version 2.5.1 (http://www.R-project.org) and the *gcrma* and *limma* libraries of Bioconductor version 2.0 (http://www.bioconductor.org). Expression values were computed using *gcrma* [25]. Differential expression of genes was determined using an empirical Bayes approach within *limma* [47], with the factor “genotype” (wild-type, *Nurf301* Null, *Nurf301 ΔC*). Moderated *t* statistics based on shrinkage of the estimated sample variance toward a pooled estimate and the corresponding *P* values were calculated for the *Nurf301* Null vs. wild-type, and *Nurf301ΔC* vs. wild-type comparisons. *P* values were adjusted according to Benjamini and Hochberg [48] to control the false discovery rate and a threshold of 0.01 was used to select probe sets. Probe-sets with statistically significant changes relative to wild-type were determined using the *decideTests* function in *limma*. GCRMA computed expression values for all genes are listed in Dataset S1. The top 500 and 200 genes with altered expression in respectively the null *Nurf301* and *Nurf301ΔC* mutants are listed in Dataset S2 and Dataset S3. Array datasets are available through ArrayExpress (accession number E-MEXP-2011).

### Semi-quantitative RT–PCR analysis

For confirmation of microarray expression data, mRNA was isolated from whole larvae as described above and reverse transcribed by Superscript II (Invitrogen) at 42°C. PCR was performed for 25–32 cycles as described [13]. Primer sets used are listed in Table S2. For analysis of testis gene expression testes were dissected from wild-type and *Nurf301^4^* homozygous mutant 3–5 day old male flies in ice-cold HyQ-CCM-3 culture medium (HyClone) containing protease inhibitors (Complete, Roche). Samples of 25 pairs of testes were then centrifuged and washed in ice-cold 1×PBS containing protease inhibitors. Testes pellets were stored at −80°C until use. mRNA was isolated using mMacs columns as described above and reverse transcribed as above. Primer sets used for PCR are listed in Table S3.

### Western analysis

Imaginal discs were dissected from 40 third instar larvae of each genotype. Samples were homogenized in SDS-PAGE loading buffer and run on 6% SDS-PAGE gels. Western analysis was using Rabbit anti-NURF301 ([15]; 1∶1000) and anti-β-Tubulin antibodies (MAb E7; 1∶100).

### Immunofluorescence and live imaging

Testes were dissected from 3–5 day old male flies in ice-cold HyQ-CCM-3 culture medium (HyClone) containing protease inhibitors. Testes were fixed in PBT (PBS containing 0.1% Triton-×100 (Sigma)) containing 3.7% paraformaldehyde (Sigma) for 25 min on ice. Samples were washed in PBT, blocked in blocking solution (PBT containing 10% FCS) for 1 hour at room temperature. Primary antibody incubation was performed in blocking solution for 3 hours at room temperature. Mouse MAb F2F4 (anti-Cyclin B) was used at the dilution 1∶5. Cy3-conjugated secondary antibodies (Jackson ImmunoResearch) were used at 1∶1000 in PBT for 1 hour at room temperature. Samples were mounted in Vectashield containing DAPI (Vector Laboratories). For phase microscopy of live testes, testes were squashed in testes buffer (183 mM KCl, 47 mM NaCl, 10 mM Tris (pH 6.8)) containing 4 µg/ml Hoechst 33342). Samples were observed using a confocal microscope (Zeiss Axiovert 100 M). For anti-NURF301 and anti-H3K4me3 staining testes were dissected in testis buffer, fixed in Hepes buffer (0.1 M Hepes, 2 mM MgSO_4_, 1 mM EGTA (pH 6.9)) containing 3.7% paraformaldehyde for 12 minutes at room temperature and squashed under a siliconized coverslip. Coverslips were removed after freezing in liquid nitrogen and slides stored in PBTw (PBS containing 0.1% Tween-20 (Sigma)) until use. Blocking and primary antibody incubation was performed in PBTw containing 10% FCS at 4°C overnight. Washes and secondary antibody incubation was performed in PBTw at room temperature as described above. Anti-NURF301 [15] and anti-H3K4me3 antibodies (Abcam, Ab8580) were used at 1∶1000 and 1∶2000 respectively. Slides were mounted and viewed as above.

### Chromatin immunoprecipitation (ChIP)

Testes were dissected in batches of 25 from 3–5 day old male flies in ice-cold HyQ-CCM-3 culture medium (HyClone) containing protease inhibitors. Testes were fixed in PBS containing 1% formaldehyde at 25°C, washed three times using ice cold PBS containing protease inhibitors and pelleted after each wash by centrifugation at 260×*g* for 5 minutes at 4°. Fixed testes pellets were stored at −80°C until use. Samples were homogenized in ChIP Lysis buffer using a pellet pestle. Soluble chromatin was prepared using the protocol of the Chromatin Immunoprecipitation Assay Kit (Upstate Biotechnology). Sample corresponding to 100 pairs of testes was used for anti-NURF301 ChIP, while sample corresponding to 50 pairs of testes each was used for anti-H3K4me3 and anti-H4K16Ac ChIP respectively. Samples were pre-cleared using Protein A-conjugated magnetic beads (Dynal) for thirty minutes at room temperature, followed by incubation with antibody coated Protein A-conjugated magnetic beads (Dynal) for 2 hours at room temperature. Immune complexes were recovered by magnetic selection, washed and eluted using the protocol of the Chromatin Immunoprecipitation Assay Kit. Target DNA abundance in ChIP eluates was assayed by quantitative PCR with addition of 0.2 µCi [α-32P]-deoxyadenosine 5′-triphosphate (Perkin Elmer, specific activity 6000 Ci/mMol) as a tracer before the amplification step. The following antibodies were used rabbit anti-NURF301 [15], anti-H3K4me3 (Abcam ab8580) and anti-H4K16Ac (Serotec AHP417). Primer pairs used for PCR are listed in Table S4.

### Histone tail peptide pull-downs

The following biotinylated histone tail peptides were used: unmodified H3 (1–21) tail peptide (Upstate Biotech, 12–403); H3K4me3 (1–21) tail peptide (Upstate Biotech, 12–564); unmodified H4 (1–21) tail peptide (Upstate Biotech, 12–405); H4K16Ac (1–20) tail peptide (USBiological, H5110-15Q1). GST-PHD (NURF301 aa 2490–2553) and GST-Bromo (NURF301 aa 2525–2669) were purified as described previously [8],[17]. Peptide pull-downs were performed by incubating 1 µg tail peptide with 5 µg of either GST, GST-PHD2 or GST-Bromo protein in binding buffer (50 mM Tris, 150 mM NaCl, 0.1% NP-40) at 4°C overnight on a rotator. Complexes were incubated with 20 µL Immobilized Streptavidin (Pierce) for 2 hours at 4°C and precipitated by centrifugation at 1500 *g* for 1 min. Beads were washed four times in binding buffer and resuspended in SDS PAGE loading buffer. GST-fusion proteins were resolved on 10% SDS-PAGE gels, blotted onto PVDF membranes and visualized by Western blotting using HRP-coupled anti-GST antibodies (GE Healthcare). A 10% input lane for each fusion protein was run as a control. For the reciprocal pull-down, reactions were performed identically except Glutathione-Sepharose beads (GE Healthcare) were used for the pull-down. Peptides were resolved on Novex 10–20% Tricine Gels (Invitrogen), blotted onto PVDF membranes and visualized by staining with Streptavidin-conjugated HRP (Vectastain Elite ABC kit, Vector Laboratories). A 10% input lane for each peptide was run as a control.

### NURFΔC complex purification

NURFΔC complex was reconstituted by baculovirus-mediated co-expression in SF9 cells and purified as described previously [49]. Virus encoding FLAG-NURF301ΔC (aa 1–1557), HA-ISWI, MYC-NURF55 and AU1-NURF55 were used. NURFΔC complex was resolved on 4–20% SDS-PAGE gradient gels (Invitrogen). Association of NURF subunits with NURF301ΔC was assayed by Western blotting using anti-FLAG (Sigma, M2; 1/4000); anti-ISWI (1∶1000), anti-MYC (9E10, 1∶100) and anti-NURF38 ([50]; 1∶2000) antibodies.

## Supporting Information

Figure S1 (A) Northern analysis of 0–18 h embryo total RNA using probes covering the *Nurf301* 5′ region detects two transcripts corresponding to *Nurf301-A/Nurf301B* and *Nurf301-C* (lane 1). A probe specific to the *Nurf301-C* 3′ UTR detects only the lower band (lane 2). (B) Anti-NURF301 antibodies detect two bands corresponding to NURF301-A/B and NURF301-C in wild-type (*wt*) extracts. Both bands are lost in null *Nurf301^2^* mutant (*Null*) extracts. In *Nurf301ΔC* mutant (*Nurf301^4^*, abbreviated as *ΔC*) extracts, NURF301-A/B is lost and a truncated version of NURF301-C, NURF301ΔC, is detected. (C) Nucleotide sequence comparison of the region encompassing exon 6 and exon 7 of *Nurf301* from *Drosophila melanogaster* (D_mel), *D. pseudobscura* (D_pse) and *D. mojavensis* (D_moj). The regions corresponding to the exons in the original *Nurf301-A* cDNA are indicated by dark bars and show high nucleotide sequence conservation (asterisks) except at some wobble base pairs (the third nucleotide in a codon identified in light grey). The known intron between Exon6 and Exon7 shows low nucleotide sequence conservation. The transcript *Nurf301-C* is predicted to arise from the absence of splicing at the Exon7/Intron boundary resulting in a transcript that runs on for an extra 5 codons before a termination codon. This region shows sequence conservation typical of coding region, not intron sequence, until the termination codon after which sequence conservation is lost. This implies that this region encodes functional protein as predicted in the *Nurf301-C* transcript.
(1.25 MB TIF)

Figure S2 Real-time PCR analysis of transcript abundance in null *Nurf301* and *Nurf301ΔC* mutant 3rd instar larvae relative to wild-type larvae. Real-time PCR confirms expression changes observed using semi-quantitative RT-PCR. Transcript abundance is normalized to *rp49*.
(0.76 MB TIF)

Figure S3 Reduced Cyclin B protein levels in *Nurf301ΔC* testes. Anti-Cyclin B staining of (A) wild type and (B) *Nurf301ΔC* (*Nurf301^4^/Nurf3011^2^*) mutant testes reveals reduced Cyclin B protein levels (shown in green) in mutant primary spermatocytes. Primary spermatocytes are recognized by the tripartite nuclear structure revealed by DAPI staining (shown in red). (C) Western analysis of wild type and *Nurf301ΔC* mutant testes. 40 testes of each genotype were dissected from 3–5 day old adult male flies, homogenized in SDS-PAGE gel loading buffer and separated on 10% SDS-PAGE gels. Western analysis using antibodies against Boule (Bol, Cheng et al., 1998) and Cyclin B (sc-15872, Santa Cruz Biotech), confirms reduction of Cyclin B levels in *Nurf301ΔC* mutant testes and shows that Bol levels are unaffected in *Nurf301ΔC* mutant testes. Antibody staining of β-Tubulin (MAb E7) provides a loading control.
(2.84 MB TIF)

Figure S4 Real-time PCR analysis of testis transcript abundance. Real-time PCR confirms down-regulation of *fzo* expression in *Nurf301ΔC* mutant testes. Transcript abundance is normalized to *rp49*. (B) Blow-up of (A) to show lower fold changes.
(1.19 MB TIF)

Figure S5 Genomic interval flanking *aret* (the gene encoding Bruno), showing the location of the flanking paralog *bru-2*, and the intervening gene *CG31862*. Primer sets used in RT-PCR are indicated by arrows. Note two primer sets used for *aret* RT-PCR, one only detects the male-specific *aret-RB* transcript, the other detects *aret-RB*, *aret-RC* and *aret-RA*.
(0.17 MB TIF)

Table S1 RT-PCR primers used to detect *Nurf301* isoforms.
(0.07 MB PDF)

Table S2 RT-PCR primers used to confirm *Nurf301* mutant microarray data.
(0.09 MB PDF)

Table S3 RT-PCR primers used for analysis of testis expression.
(0.10 MB PDF)

Table S4 ChIP PCR primer pairs.
(0.07 MB PDF)
