# Supplementary figures and images for: Alternative Splicing of NURF301 Generates Distinct NURF Chromatin Remodeling Complexes with Altered Modified Histone Binding Specificities
Source: PLoS Genet. 2009 Jul 24;5(7):e1000574. doi: 10.1371/journal.pgen.1000574 (PMC2705796; doi:10.1371/journal.pgen.1000574)

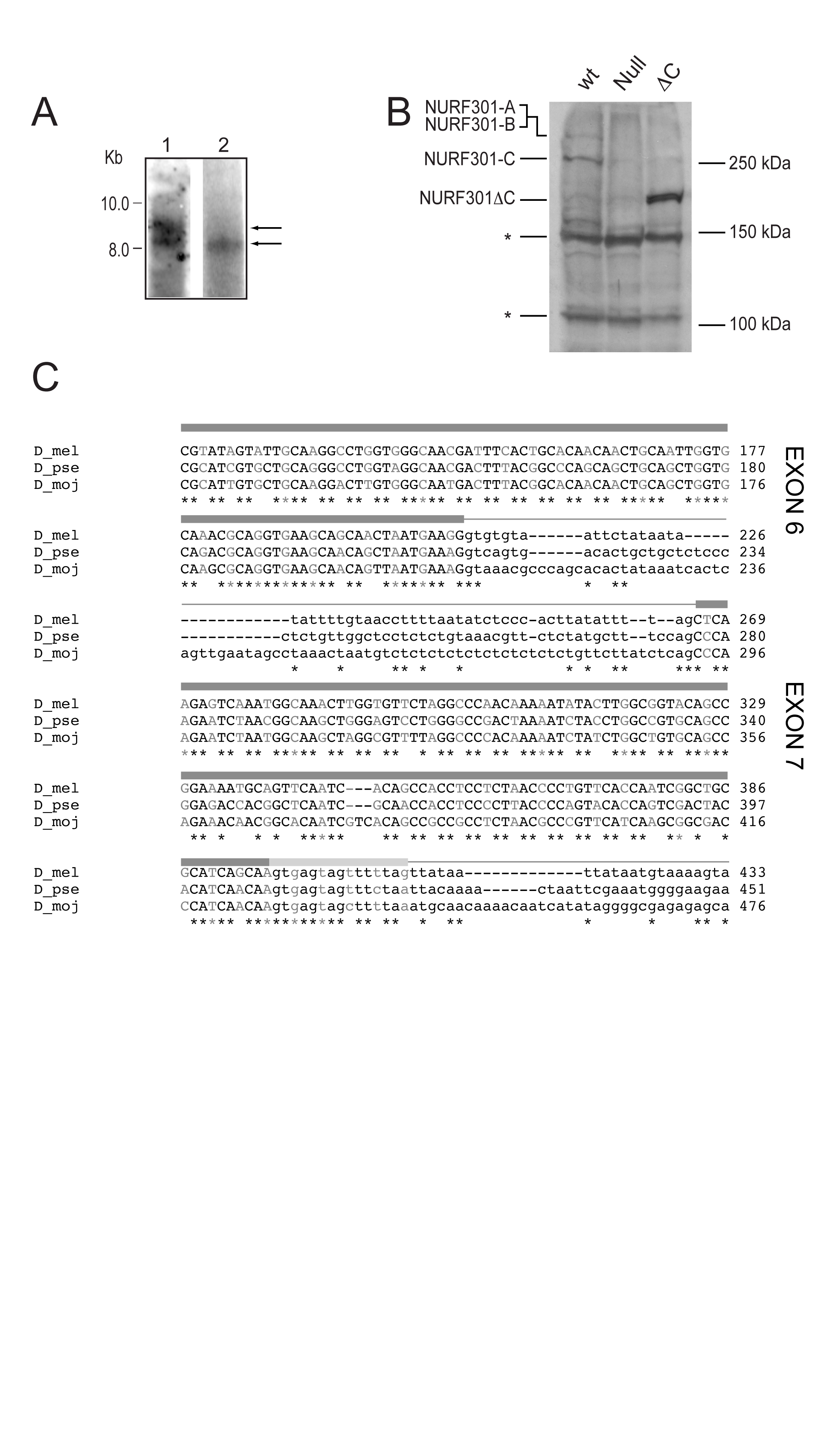

Supplement: Figure S1 — (A) Northern analysis of 0–18 h embryo total RNA using probes covering the Nurf301 5′ region detects two transcripts corresponding to Nurf301-A/Nurf301B and Nurf301-C (lane 1). A probe specific to the Nurf301-C 3′ UTR detects only the lower band (lane 2). (B) Anti-NURF301 antibodies detect two bands corresponding to NURF301-A/B and NURF301-C in wild-type (wt) extracts. Both bands are lost in null Nurf3012 mutant (Null) extracts. In Nurf301ΔC mutant (Nurf3014, abbreviated as ΔC) extracts, NURF301-A/B is lost and a truncated version of NURF301-C, NURF301ΔC, is detected. (C) Nucleotide sequence comparison of the region encompassing exon 6 and exon 7 of Nurf301 from Drosophila melanogaster (D_mel), D. pseudobscura (D_pse) and D. mojavensis (D_moj). The regions corresponding to the exons in the original Nurf301-A cDNA are indicated by dark bars and show high nucleotide sequence conservation (asterisks) except at some wobble base pairs (the third nucleotide in a codon identified in light grey). The known intron between Exon6 and Exon7 shows low nucleotide sequence conservation. The transcript Nurf301-C is predicted to arise from the absence of splicing at the Exon7/Intron boundary resulting in a transcript that runs on for an extra 5 codons before a termination codon. This region shows sequence conservation typical of coding region, not intron sequence, until the termination codon after which sequence conservation is lost. This implies that this region encodes functional protein as predicted in the Nurf301-C transcript. (1.25 MB TIF) [file pgen.1000574.s001.tif]

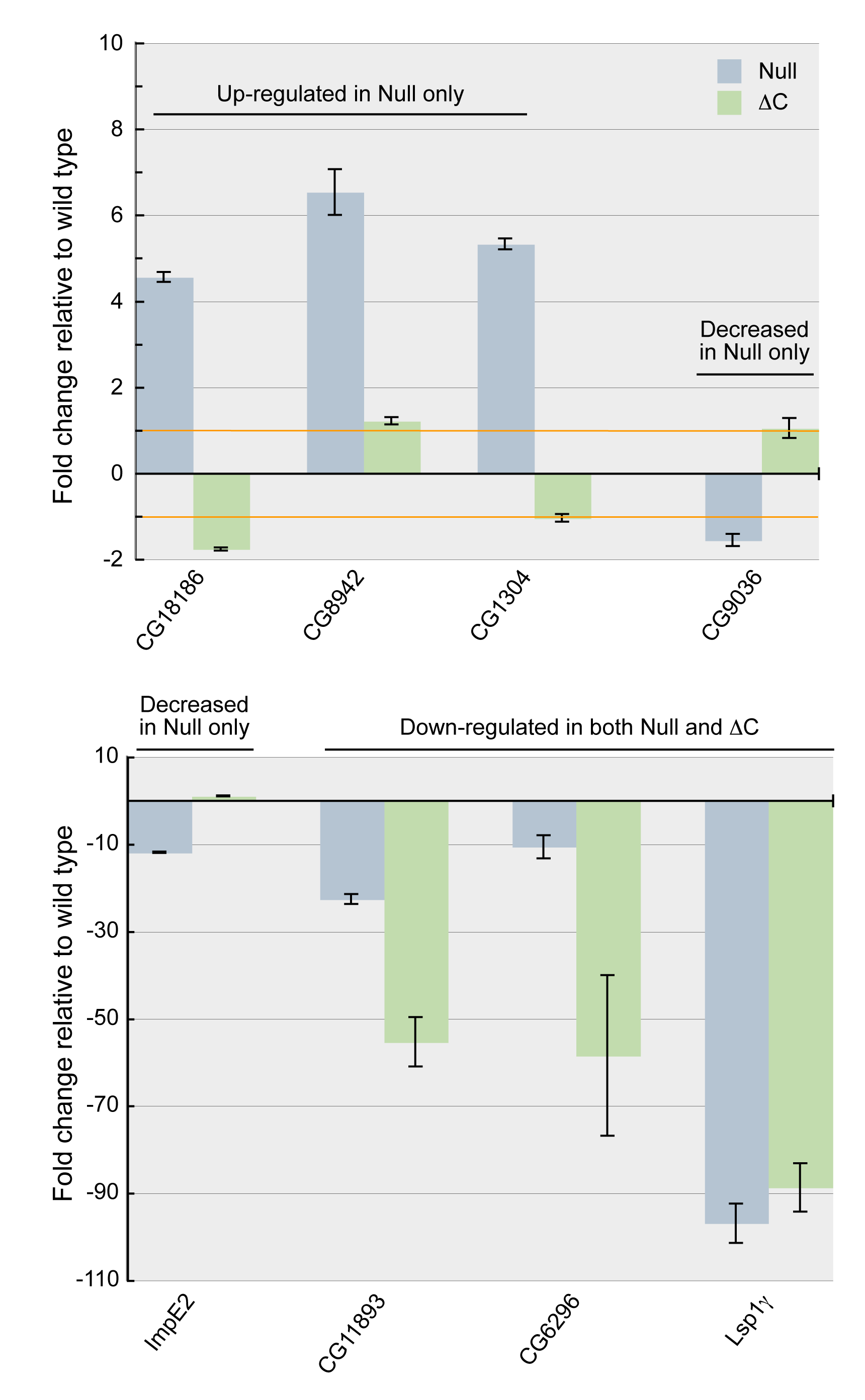

Supplement: Figure S2 — Real-time PCR analysis of transcript abundance in null Nurf301 and Nurf301ΔC mutant 3rd instar larvae relative to wild-type larvae. Real-time PCR confirms expression changes observed using semi-quantitative RT-PCR. Transcript abundance is normalized to rp49. (0.76 MB TIF) [file pgen.1000574.s002.tif]

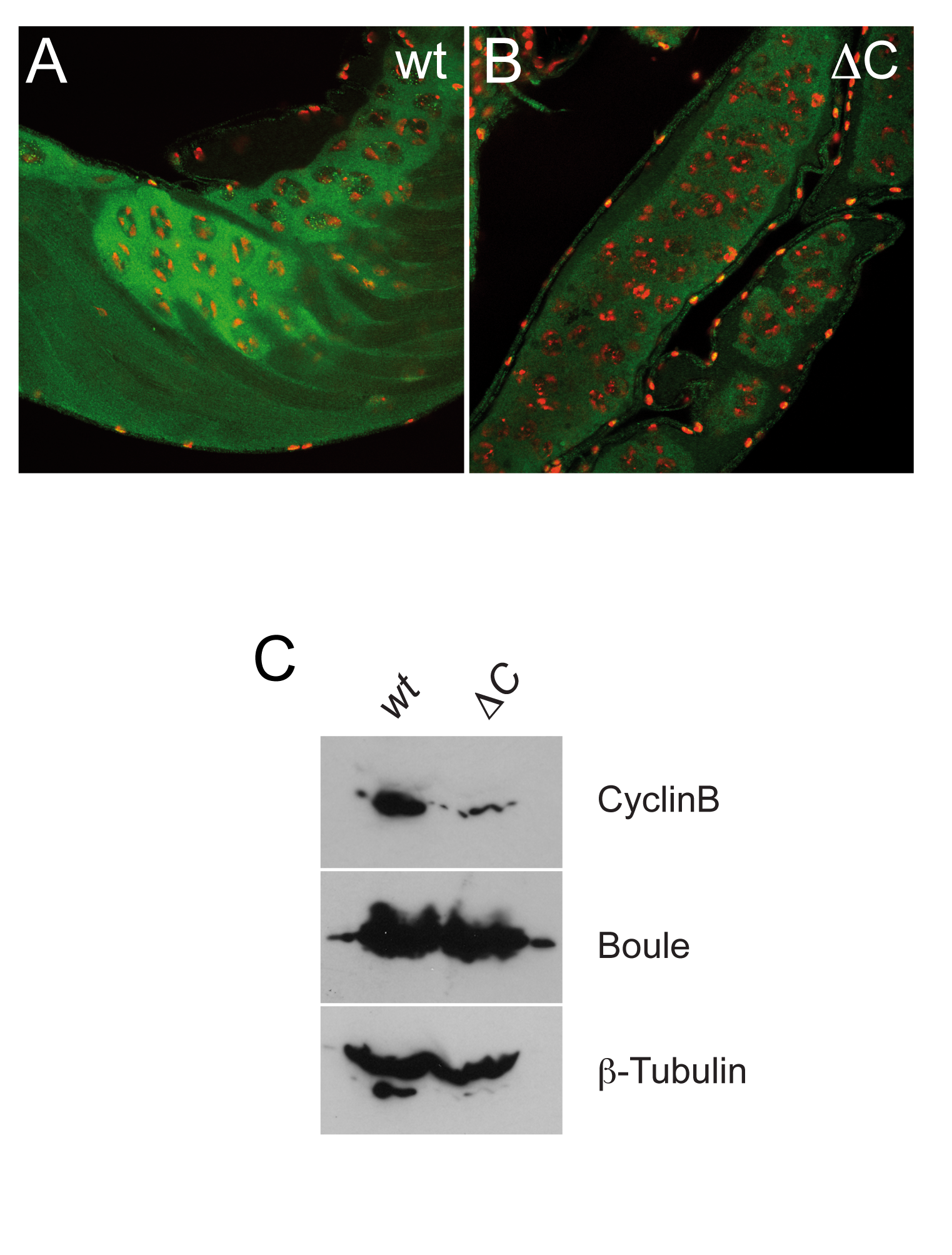

Supplement: Figure S3 — Reduced Cyclin B protein levels in Nurf301ΔC testes. Anti-Cyclin B staining of (A) wild type and (B) Nurf301ΔC (Nurf3014/Nurf30112) mutant testes reveals reduced Cyclin B protein levels (shown in green) in mutant primary spermatocytes. Primary spermatocytes are recognized by the tripartite nuclear structure revealed by DAPI staining (shown in red). (C) Western analysis of wild type and Nurf301ΔC mutant testes. 40 testes of each genotype were dissected from 3–5 day old adult male flies, homogenized in SDS-PAGE gel loading buffer and separated on 10% SDS-PAGE gels. Western analysis using antibodies against Boule (Bol, Cheng et al., 1998) and Cyclin B (sc-15872, Santa Cruz Biotech), confirms reduction of Cyclin B levels in Nurf301ΔC mutant testes and shows that Bol levels are unaffected in Nurf301ΔC mutant testes. Antibody staining of β-Tubulin (MAb E7) provides a loading control. (2.84 MB TIF) [file pgen.1000574.s003.tif]

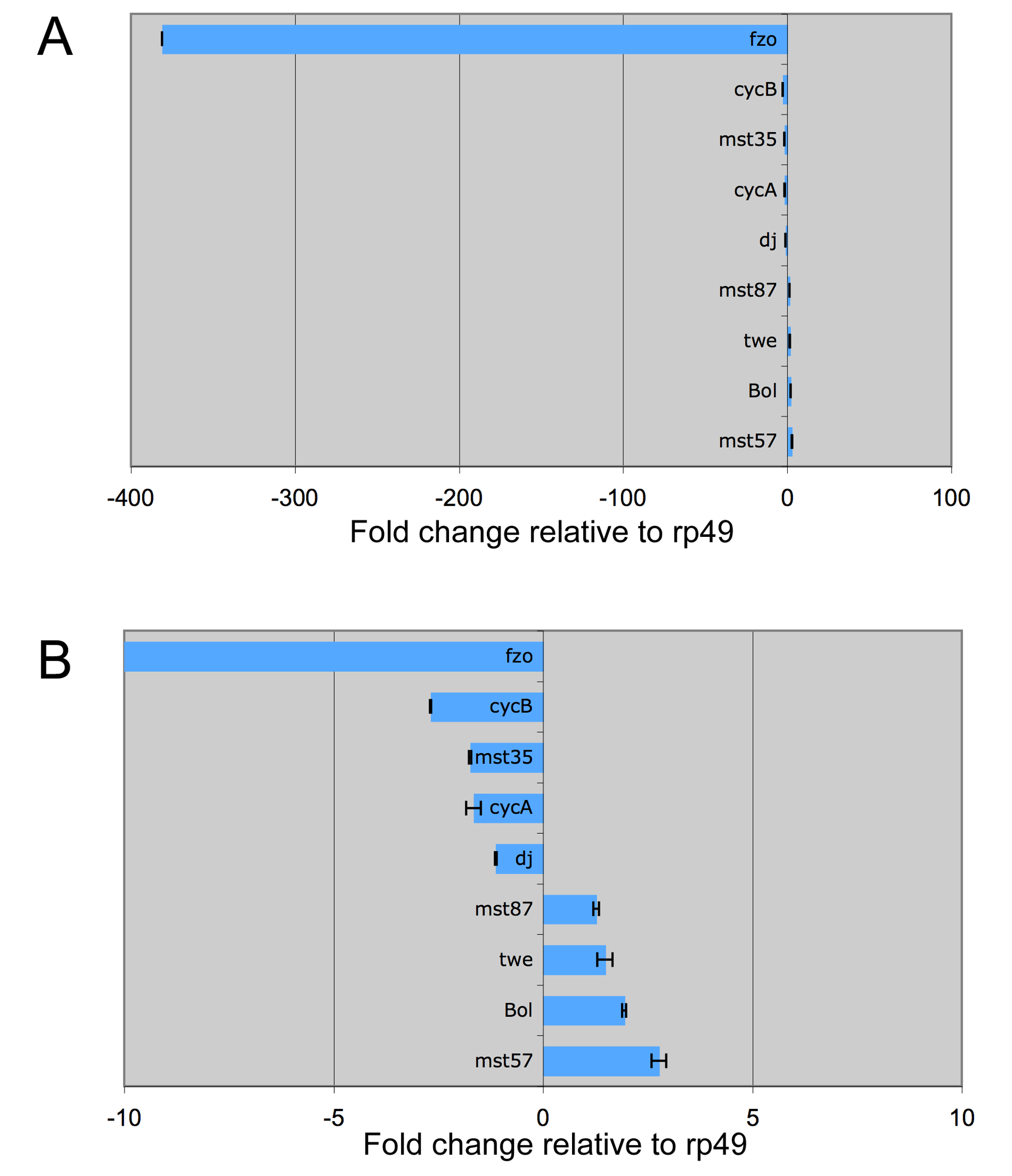

Supplement: Figure S4 — Real-time PCR analysis of testis transcript abundance. Real-time PCR confirms down-regulation of fzo expression in Nurf301ΔC mutant testes. Transcript abundance is normalized to rp49. (B) Blow-up of (A) to show lower fold changes. (1.19 MB TIF) [file pgen.1000574.s004.tif]

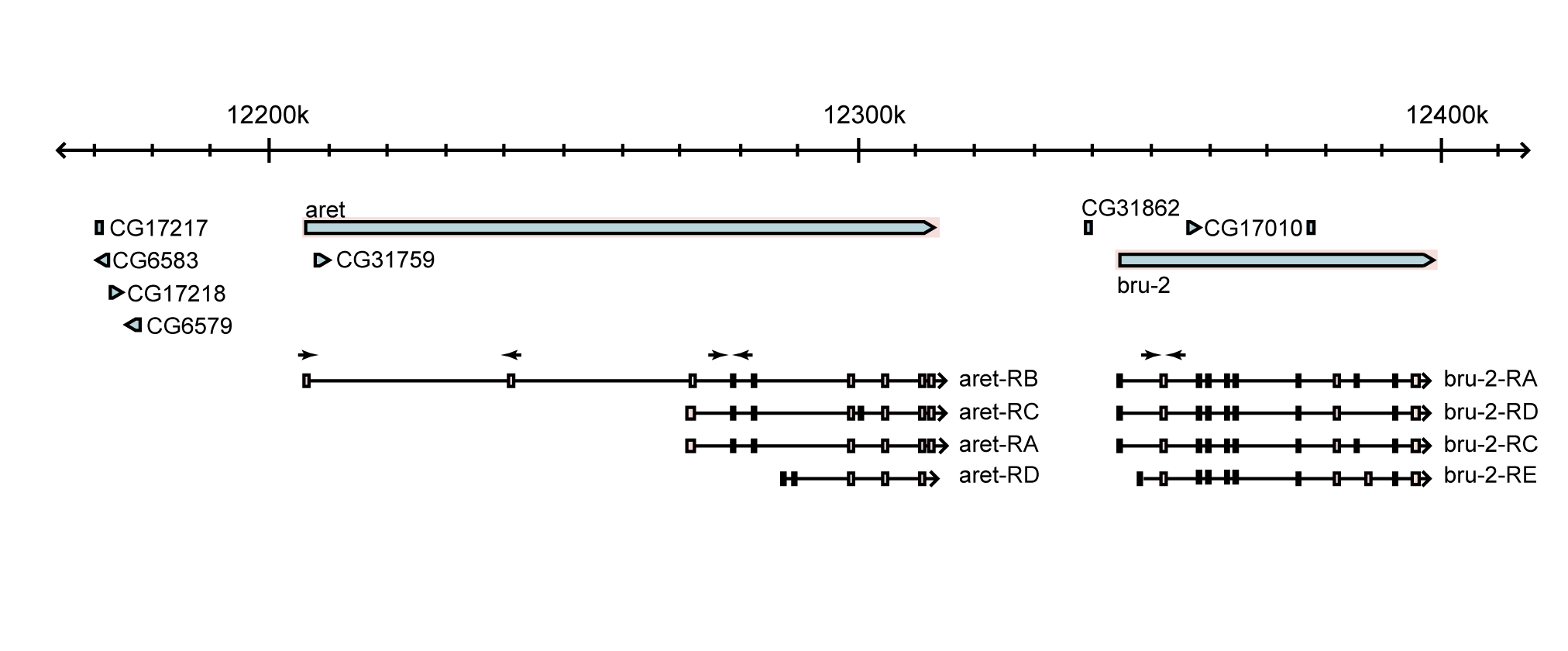

Supplement: Figure S5 — Genomic interval flanking aret (the gene encoding Bruno), showing the location of the flanking paralog bru-2, and the intervening gene CG31862. Primer sets used in RT-PCR are indicated by arrows. Note two primer sets used for aret RT-PCR, one only detects the male-specific aret-RB transcript, the other detects aret-RB, aret-RC and aret-RA. (0.17 MB TIF) [file pgen.1000574.s005.tif]
