# Supplementary material for: Alternative Splicing of NURF301 Generates Distinct NURF Chromatin Remodeling Complexes with Altered Modified Histone Binding Specificities
Source: PLoS Genet. 2009 Jul 24;5(7):e1000574. doi: 10.1371/journal.pgen.1000574 (PMC2705796; doi:10.1371/journal.pgen.1000574)
Supplement: Table S1 — RT-PCR primers used to detect Nurf301 isoforms. (0.07 MB PDF) [file pgen.1000574.s006.pdf]

Table S1

RT-PCR primers used to detect *Nurf301* isoforms

Isoform	***Primer name***	***Primer sequence (5' to 3')***	***Product length (bp)***
Nurf301-RA	AF	TCGGTAATCAACCAACGCAA	1105
	ABR	AGTTGTTGCTAGCGTATCGG	
Nurf301-RB	BF	CATGACACAGCAGCAGATAG	1056
	ABR	AGTTGTTGCTAGCGTATCGG	
Nurf301-RC	CF	TGCGCATCAGCAAGTGAGTAG	208
	CR	CGAAACGTATAGAAAGTGCAAGTG	
