# Supplementary material for: Alternative Splicing of NURF301 Generates Distinct NURF Chromatin Remodeling Complexes with Altered Modified Histone Binding Specificities
Source: PLoS Genet. 2009 Jul 24;5(7):e1000574. doi: 10.1371/journal.pgen.1000574 (PMC2705796; doi:10.1371/journal.pgen.1000574)
Supplement: Table S2 — RT-PCR primers used to confirm Nurf301 mutant microarray data. (0.09 MB PDF) [file pgen.1000574.s007.pdf]

Table S2

RT-PCR primers used to confirm *Nurf301* mutant microarray data

Gene	*Primer name*	*Primer sequence (5' to 3')*	*Product length (bp)*
Lsp1γ	Lsp1γ_5P	AGGAGCGCCTGGCCAACGGT	200
	Lsp1γ_3P	GGACCTTGTAGACGCGACTG	
CG6296	CG6296_5P	GCTTGGTTCCAGTACGGCGA	179
	CG6296_3P	GAAGCCCACAATTTCCAGAG	
CG11893	CG11893_5P	GCCGATGAGCTGGAGGCTCC	166
	CG11893_3P	ACTCAGCAGTAGTACGGAAC	
ImpE2	ImpE2_5P	GATCCTGGTTTTCCTGGCCATTAGCCAAGC	519
	ImpE2_3P	CTCGGCAGGCACAACGACAGTCTCTTCAGC	
CG9036	CG9036_5P	GCCCACGCAGGGCTACCAGTCGCCGTCGAG	479
	CG9036_3P	CCGCCACCGTTGCGACCATTGCCATTGCCG	
CG1304	CG1304_5P	CCCTGAAGTCCATTTCCTTG	193
	CG1304_3P	TCCATCTGGATAGCTCGTTC	
CG8942	CG8942_5P	ACTGCAGACATCAACCAGCGAGGTTCCGCA	429
	CG8942_3P	TTCTTCGGCTTCAAAATTTCGCCTAGAGGT	
CG18186	CG18186_5P	GTGTCCGAAACGTCAAGGAG	199
	CG18186_3p	GCTCTGCTGGAATAGTTCGG	
rp49	rp49_5P	TCTTGAGAACGCAGGCGACCGTTGGGGTTG	337
	rp49_3P	ATCCGCCACCAGTCGGATCGATATGCTAAG	
