# Supplementary material for: Alternative Splicing of NURF301 Generates Distinct NURF Chromatin Remodeling Complexes with Altered Modified Histone Binding Specificities
Source: PLoS Genet. 2009 Jul 24;5(7):e1000574. doi: 10.1371/journal.pgen.1000574 (PMC2705796; doi:10.1371/journal.pgen.1000574)
Supplement: Table S3 — RT-PCR primers used for analysis of testis expression. (0.10 MB PDF) [file pgen.1000574.s008.pdf]

Table S3

RT-PCR primers used for analysis of testis expression

Gene	*Primer name*	*Primer sequence (5' to 3')*	*Product length (bp)*
arrest	aret-5P	GTCCCATACACAGCAACAAC	162
	aret-3p	CCGTTGCTGCCTACGGAAAC	
arrest-mst	aret-mst-5P	CCAGTTATCCGCTTTCGGC	154
	aret-mst-3P	TGTGGTCTCCTTACTTTGG	
bol	bol_5P	ATCTTTGTGGGTGGCATCAGC	213
	bol_3P	CAATGTTCAGCTTCCGATCTC	
bruno-2	bru2-5P	GAGTCTGCACGCGGATAAAC	153
	bru2-3P	GCCATTAGCAACATGGGCAAC	
CG17218	CG17218-5P	CCGGCAGAAGGTGCACGGTG	159
	CG17218-3P	CGAGTTGCAGCCGTCCTTGC	
CG31862	CG31862-5P	CAACTAGCGAGCCAGTGATC	173
	CG31862-3P	GTCGCTCTTCATCGCTGAAG	
cyclin A	cycA_5P	CAAATGGCGGTGGTGCGCTC	199
	cycA_3P	GCAGTCGGTGTGCACAGATC	
cyclin B	cycB_5P	GAGGACAGCAATCTGTCGAAG	221
	cycB_3P	GGTCGTAGTGACTGCACTGT	

don juan	dj_5P	CGGCCTCACCACATCAATGT	150
	dj_3P	CTTGCAAGAACTTTCGTTGC	
fuzzy onions	fzo_5P	GTAATTACCTGTCCAATTTC	142
	fzo_3P	TGCCACCTTCATTCGATCAC	
mst35B	mst35B_5P	TGTGCCAAGGCGACTCGGCG	177
	mst35B_3P	CTTTGCAATTAATTCCCGCG	
mst57B	mst57B_5P	ACGTGGCAAAGGCCGAAAGA	146
	mst57B_3P	CACTCCTTCCGGTTTCCAAG	
mst87F	mst87F_5P	CGAATTAATCATGTGCTGCG	149
	mst87F_3p	CCACAGGGTCCACAGGCATA	
twine	twi_5P	CAGCACCACCGTTCTGTCGC	139
	twi_3P	GTGTTCGCGAGGACAGGCTA	
rp49	rp49_5P	ATCCGCCACCAGTCGGATCGATATGCTAAG	337
	rp49_3P	TCTTGAGAACGCAGGCGACCGTTGGGGTTG	
