# Supplementary material for: Alternative Splicing of NURF301 Generates Distinct NURF Chromatin Remodeling Complexes with Altered Modified Histone Binding Specificities
Source: PLoS Genet. 2009 Jul 24;5(7):e1000574. doi: 10.1371/journal.pgen.1000574 (PMC2705796; doi:10.1371/journal.pgen.1000574)
Supplement: Table S4 — ChIP PCR primer pairs. (0.07 MB PDF) [file pgen.1000574.s009.pdf]

Table S4

ChIP PCR primer pairs

Gene region	***Primer name***	***Primer sequence (5' to 3')***	***Product length (bp)***
fzo p1	fzo-p1_5P	GTTGTACTTCCGAAGTGTGC	210
	fzo-p1_3P	CCGAAGGGCAAACACAGCGG	
fzo p2	fzo-p2_5P	TCCATGATTGCGGTAATTGTG	212
	fzo-p2_3P	CTTAGTTCAATGGGCATGCAA	
fzo p3	fzo-p3_5P	TTAATGTGTCACAACCCCTC	123
	fzo-p3_3P	AGGACACCGACGACGTACTT	
fzo p4	fzo-p4_5P	CAGCGCCATGGGCCATACCA	134
	fzo-p4_3P	TGTGCACTGGCCAGTTGGCT	
fzo p5	fzo-p5_5P	GAAGCCCAGTCCAATGAGAC	199
	fzo-p5_3P	GGTCTCTTGAAAGTCTC	
